# An Odor Timer in Milk? Synchrony in the Odor of Milk Effluvium and Neonatal Chemosensation in the Mouse

**DOI:** 10.1371/journal.pone.0047228

**Published:** 2012-10-25

**Authors:** Syrina Al Aïn, Laurine Belin, Bruno Patris, Benoist Schaal

**Affiliations:** Developmental Ethology and Cognitive Psychology Group, Center for Smell, Taste and Food Science, CNRS (UMR 6265), Université de Bourgogne, Dijon, France; Barnard College, Columbia University, United States of America

## Abstract

Mammalian newborns exhibit avid responsiveness to odor compounds emanating from conspecific milk. Milk is however developmentally heterogeneous in composition as a function of both evolved constraints and offspring demand. The present study aimed to verify whether milk odor attractivity for neonates is equally distributed along lactation in *Mus musculus* (Balb-c strain). Therefore, we exposed pups varying in age to milk samples collected from females in different lactational stages. The pups were assayed at postnatal days 2 (P2), 6 (P6) and 15 (P15) in a series of paired-choice tests opposing either murine milk and a blank (water), or two samples of milk collected in different stages of lactation [lactation days 2 (L2), 6 (L6), and 15 L15)]. Pups of any age were able to detect, and were attracted to, the odor of the different milk. When milk from different lactational stages were simultaneously presented, P2 pups oriented for a similar duration to the odors of L2 and of L6 milk, but significantly less to the odor of L15 milk. Next, P6 pups roamed equivalently over L2 and L6 milk odors, but still less over the odor of L15 milk. Finally, P15 pups explored as much L15 milk odor as the odors of both L2 and L6 milk. This developmental shift in milk attractivity is discussed in terms of changing chemosensory properties of milk and of shifting chemosensory abilities/experience of pups.

## Introduction

Newborns from any mammalian species studied so far show avid responsiveness to odor cues emitted by mammary structures, especially in milk or mammary secretions ([1]–[2] for reviews). Milk is indeed affluent in odor-active compounds [3]–[6]. But it is also constantly changing in composition and its olfactory profile is expected to fluctuate accordingly. In addition to individual variations linked with a female's genetic background and habitus (ecological niche, diet), milk composition is indeed physiologically determined along several time scales: *i.e.*, the female's reproductive stage (parity, co-occurring lactation and pregnancy), lactational phase (colostral, transitional, mature milk), time of day (morning *vs.* evening milk), and the nursing cycle (fore- *vs.* hindmilk). Evidence for compositional milk variability over time comes mainly from studies on macro- and micronutrients, and bioactive peptides [7]–[9], but very seldom from studies on the chemosensory correlates of such constituents or on how newborns react to them. So far, the evidence for neonatal responses to time-bound sensory properties of milk is meager and controversial [10]–[14].

Covariation between milk odor and infant perception supposes that milk composition is reflected in its odor/flavor properties and that neonatal organisms can detect them. The nutritional, immunological and chemocommunicative requirements of newborns changing over the course of development, and the composition of milk being adjusted accordingly [15], [16], an offspring's ability to discriminate between females that are in different lactational stages, or between their milk, may promote the intake of developmentally-appropriate nutritive and protective elements, especially in the case of species practicing shared suckling or communal nursing.

The present study aimed to address whether there are periods in lactation during which milk would carry stronger olfactory potency for newborns. It was run in the mouse because newborn pups were shown to respond to conspecific milk odor [17] and because mouse milk compositional fluctuations are known during the first days of lactation and between weeks 1 and 2 of lactation [18]–[22], assuming odorous metabolites would change correlatively. To assess neonatal responses to hypothetic fluctuations in milk odor, we used a nested method in exposing infant mice of varying ages (potentially changing in olfactory abilities) to milk collected from females in different lactational stages (potentially varying in odor properties).

The paucity of related data from other mammalian taxa provides a poor heuristic ground for predictions. However, it might be suggested that behaviorally-potent odorants, if any, should be emitted in higher concentration when the selective constraints on offspring are maximal. In this case, mouse pups from any age should react more avidly to early-lactation than to late-lactation milk. A second possibility assumes a degree of adaptive matching between the biological impact of milk, its sensory properties, and offspring chemoreceptive selectivity. Accordingly, mouse pups might olfactorily react more to a milk matched with own age because of its beneficial interoceptive impact. A third possibility would suggest that pups do positively react to any milk from previous lactational stages because they have got opportunities to sense and learn them. Finally, as maternal behavior of *Mus musculus* involves communal nursing [23], [24], an opportunistic strategy might be adaptive, and non-age-selective responsiveness to milk odor might be envisioned accordingly. There is no *a priori* ground to privilege one among the above hypotheses, and the present work is intended to begin sorting them out based on behavioral assays.

## Results

### Attraction towards the odor of milk from different lactational ages

**Figure 1:**
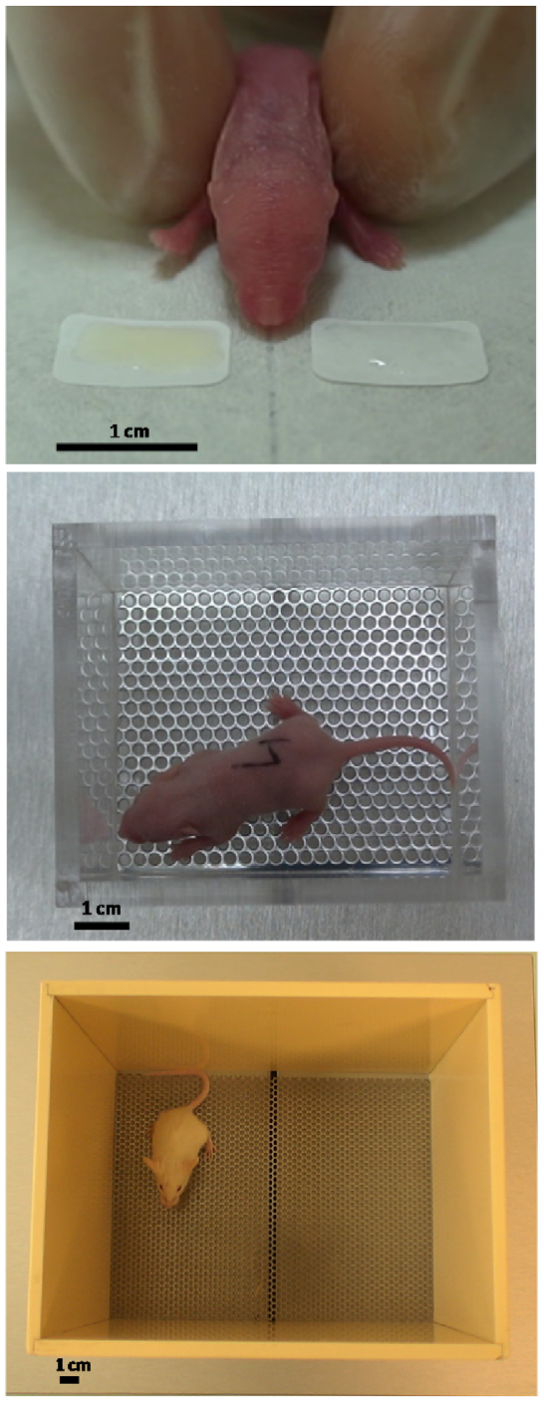
Absolute attraction to milk odors from different lactational stages against the control stimulus. Box plot of the duration 2, 6, and 15 day-old (P2, P6 and P15) mouse pups spent oriented to: 1) milk of lactation day 2 (L2) *vs.* water (W), 2) milk of lactation day 6 (L6) *vs.* water, and 3) milk of lactation day 15 (L15) *vs.* water. The dashed line indicates the theoretical level of random orientation. Wilcoxon's tests: ** p<0.01, * p<0.05. The central square within the box represents the median; the box encloses the interquartile range; whiskers show 1.5 times the interquartile range, “o” indicate outliers.

As shown in Figure 1, P2 pups turned their muzzle significantly longer to the odor of L2, L6 and L15 milks than to the control stimulus (L2: Z = 2.557, p = 0.011; L6: Z = 2.763, p = 0.006; L15: Z = 1.672, p = 0.048; respectively). Likewise, P6 pups oriented significantly longer to L2, L6 or L15 milk odor than to water (L2: Z = 2.501, p = 0.012; L6: Z = 2.461; p = 0.014; L15: Z = 2,308, p = 0.021). Finally, P15 pups stayed significantly longer over L2, L6 or L15 milks than over the control stimulus (L2: Z = 2.053, p = 0.040; L6: Z = 2.029, p = 0.042; L15: Z = 2.143, p = 0.032). In sum, when presented against the control stimulus, the odor of milk from any lactational stage was detected by, and attractive to, pups of all the ages considered.

### Relative attraction towards milk odors from different lactational stages

**Figure 2:**
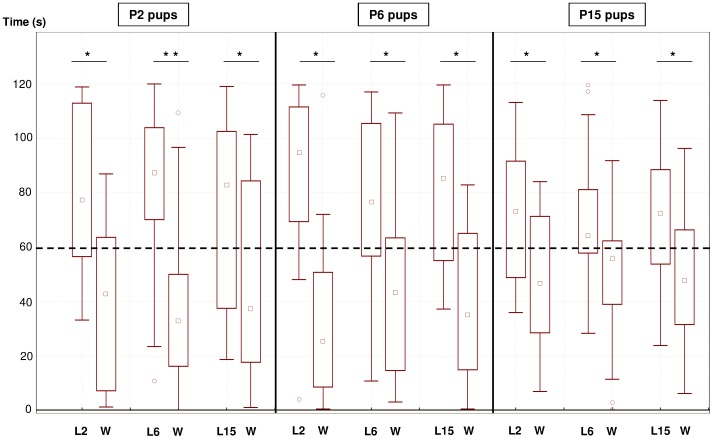
Relative attraction to the odors of milk samples from different lactational stages. Box plot of the duration 2, 6, and 15 day-old (P2, P6 and P15) mouse pups spent oriented to: 1) milk of lactation day 2 (L2) *vs.* milk of lactation day 6 (L6), 2) milk of lactation day 2 (L2) *vs.* milk of lactation day 15 (L15), and 3) milk of lactation day 6 (L6) *vs.* milk of lactation day 15 (L15). The dashed line indicates the theoretical level of random orientation. Wilcoxon's tests: ** p<0.01, * p<0.05. The central square within the box is the median; the box encloses the interquartile range; whiskers show 1.5 times the interquartile range, “o” indicate outliers.

Here, we examined whether there are periods in lactation during which milk would be more or less olfactorily attractive to P2, P6 and P15 pups (Figure 2). When exposed to the odors of L2 and L6 milk, P2 pups oriented equivalently (Z = 0.689, p = 0.485). However, in the test opposing L2 milk odor and L15 milk odor, pups oriented significantly longer to the former than to the latter (Z = 2.728, p = 0.006). Likewise, in the test opposing L6 and L15 milk odors, the pups spent more time exploring the former than the latter (Z = 2.138, p = 0.033). In sum, L2 and L6 milks bear properties that confer them equivalent attraction in P2 pups.

P6 pups roamed indiscriminately over L6 milk and L2 milk odors (Z = 0.330, p = 0.741). But, these pups oriented significantly longer to the L2 and L6 odors than to the L15 milk odor (Z = 2.324, p = 0.020 and Z = 2.053, p = 0.040, respectively). To sum up, L2 and L6 milk odors appear chemosensorily or motivationally equivalent, while both of these milks are treated differentially from L15 milk.

Finally, P15 pups explored for equivalent durations the odor of L2 milk and the odors of either L6 milk (Z = 1.531, p = 0.126) or L15 milk (Z = 0.030, p = 0.976). In addition, the odors of L6 and L15 milk were explored for similar durations (Z = 0.187, p = 0.852). Thus, P15 pups do positively react to conspecific milk odor regardless of the donor female's lactational stage.

## Discussion

The present study showed first that mouse pups of any age can detect the odor of conspecific milk, and that they are attracted to it against water regardless of the lactation stage of the donor female. This confirms previous work on the salience of murine milk for mouse pups [17], and extends the pan-mammalian phenomenon of neonatal attraction toward the odor of conspecific milk (*e.g.*, [4], [14], [25]–[27]; reviewed in [2]).

But the most significant result pertains to the neonates' *relative response* to the odor of milk samples collected at different lactation stages. Although all milk odors proved attractive in the absolute preference tests, some were more attractive than others in relative tests. These tests revealed that pups aged 6 days or below display reduced attraction to the odor of milk collected somewhere after postpartum days 6. Such a developmental shift in responsiveness may be explained either by lactation stage-related variations in the odor properties of milk, by variations in chemosensory abilities of mouse pups, or by both.

Regarding lactation stage-related variations in milk odor, we are unaware of any study that has analyzed milk composition changes along lactation in terms of *odor* or *flavor* correlates in the mouse. But numerous studies point to lactation stage-related composition changes of murine milk in terms of macronutrients and bioactive constituents. For example, lipid composition is altered between L3 and L14 [18]–[20], lactose augments until L18 [18], ε- and α_S1_-casein increase from early (L0–L2) to mid/late (L8–18) lactation [28]–[30], while the epidermal growth factor peaks around L6 and decreases at L12 [31]. Lactational changes in milk composition are obviously more extensively documented in other species, namely humans and dairy animals [9], and demonstrate species-specific developmental patterns of milk composition.

Whether and how such developmental patterns of milk composition correlate with milk odor profiles is so far unknown. Several processes translating milk composition into odor cues may be effective separately or conjointly. First, the milk content in odor-active compounds or in odor precursors may change along lactation. One such case is noted in the rabbit, in which a milk compound with high odor impact, the mammary pheromone 2-methylbut-2-enal, is significantly more concentrated in early (L3) than in late (L23) milk [32]. Second, changes in the odor-binding properties of milk may alter the profile of hydrophobic or hydrophilic volatiles. For example, minute variations in fat or protein content in a milk-based mixture notably affect the release of aroma compounds [33]–[35]. Finally, there is evidence that newborn mammals can actually detect odor correlates of changing milk composition, namely during the transition between colostrum and transitional milk [1], [36]. In sum, biochemical variations in milk might be olfactorily detected by offspring, adding communication to the recognized functions of milk that include hydration, nutrition, immunoprotection, endocrine control, and nonpathogenic bacterial transfer [1], [2], [37]–[39].

Some concern might be raised about possible consequences on milk odor of the milking procedure used here which implied anesthetizing donor females and boosting their milk ejection by oxytocin (as recommended by murine milk experts [40], [41]). Some studies demonstrated that oxytocin, xylazine and ketamine are potentially transferred into milk in dairy species [42]–[46]. However, as the same, low doses of these agents were used in all females, we consider this potential influence to be controlled across groups. This is further supported by the fact that all milk gave off odors that were attractive against water to pups of any age, and that were differentially attractive when tested between milk.

As regards age-dependent variations in chemosensory abilities of mouse pups, the youngest mice (P2, P6) approached more the odor of milk collected in earlier (L2, L6) than in later lactation (L15). Thus, L2–L6 milk may bear similar volatile profiles, which may then alter in L15 milk. P2 pups could not learn the odor of L6 milk, although compounds from L2 milk may occur in L6 milk. Similarly, P6 mice were familiarized to both early (L2, L6) milk, but not to the supposedly novel L15 milk. Learning is more evident in P15 mice that were attracted to any milk odor. Their indiscriminant responses may be due to reduced selectivity to milk odor variations either: 1) because they have already recorded beneficial consequences with all of them, or 2) because they were more susceptible to pre-test nursing deprivation leading to a higher hunger state. However, this latter possibility is mitigated by the fact that pups of all ages displayed similar levels of attraction to milk odor in the absolute tests. In sum, the present data can be interpreted in terms of exposure and learning effects which increase with age. However, for the youngest pups (P2–P6), interpretations based on fetal exposure and/or on predisposed stimulus-response coupling for some milk compounds cannot be excluded.

How do the four predictions outlined in the Introduction accommodate with the present data? The first proposal was that behaviorally-active odorants might be predominantly emitted during early lactation when neonates face maximal survival challenges. Illustrated in the rabbit (*cf.* above [4], [32]), a similar process might be involved in the responses of P2 and P6 mice in their greater attraction to the odor of early-lactation milk. A second adaptive mechanism posited that neonates could express a “bias” or an “expectation” in favor of the odor profile of age-matched milk. Although unrelated with milk, a remarkable case of female chemoemission-offspring chemoreception synchrony was shown in the rat: dams begin emitting unidentified odor agents in their soft feces at P14, a time when their pups precisely begin reacting to them, and by P28 they cease emitting this compound when the pups reduce responding to cæcotrophes [47]. Coming back to mammary function, such a matching process between milk odor and offspring chemosensory proclivities was shown in the rabbit (*cf.* above [32]) and the rat. Rats aged 12 h to 10 days grasp indeed more rapidly the nipples of a female which lactation stage is aligned with that of their own mother than the nipples of females whose lactation age differed from their own mothers' by at least 7 days [48]. Recent evidence from the rat shows that a small subset of olfactory receptor genes are expressed or overexpressed at birth, suggesting the possibility of specialized odor reception mechanisms in relation with the mother or milk [49]. The third prediction assumed that mouse pups would prefer any milk collected from females that are in the same or preceding lactational stages than that of their own mother because corresponding odor profiles could be learned [50]–[53]. This is clearly verified here in P15 (for L2, L6, and L15 milk odors) and in P6 mouse pups (for L2 and L6 milk odors). Finally, it was predicted that mouse pups would accept any murine milk in the context of the communal nursing regimen typical of *Mus musculus* [23], [24], [54]. This prediction might not hold for P2 and P6 pups when confronted with two females differing in lactational age (assuming that lactation stage-dependent differences in milk odor co-vary with odor contrasts on the females' ventrum and nipples; cf. [48]). These latter pups behaved indeed differentially in the relative attraction tests as a function of lactation stage. However, P15 pups appeared less selective in such conditions and might therefore take advantage of any lactating female in a communal nursing context. If the discrimination towards milk odors of differing lactational ages extends to the body odor of lactating females, one may predict that young pups will behave more discriminately toward allied females in the communal nursing situation than older young. This needs to be ascertained, however.

## Conclusions

Mouse neonates are differentially attracted to milk from different lactational stages, when milk odors are presented in relative attraction tests. The selective response is clearest when cumulative exposure to milk is minimal, younger mice being more attracted to milk that are matched or nearly-matched to the lactational stage of their own mother. At later ages, with expanding energetic needs and autonomy in foraging, mouse pups develop more opportunistic responses and their attraction to milk odor seems blurred by learning and generalization. In a broader view, these results highlight a possible temporal coordination in the interactions between maternal lactation physiology and neonatal perception and behavior. They raise issues about the feeding of neonates born at atypical timing (*viz.*, premature infants), and whose initial adaptation and growth might be optimized when given age-adapted milk. Although no chemosensory preference data are at hand as yet in these infants, there is evidence for more optimal development when they are fed own mothers' milk [55]–[57], which certainly is the most timely for them.

## Methods

### Ethic Statement

The French national and local rules concerning the use of laboratory animals were strictly followed. The protocol was approved by the Institutional Animal Care and Use Committee of the University of Burgundy (Protocol N° 20-09; Authorization of experimentation n° 21 CAE 059). To maximize and standardize their responsiveness, the pups were separated from the mother for 4 hours before testing. During that pre-test separation, the male was kept with the litter, and the cage was put on a heating plate (temperature fixed at 38°C), to limit potential effects of social stress and hypothermia. To prevent thermoregulatory stress, all assays were performed on another heating plate which temperature was set at 33°C. Females were milked to obtain fresh milk. Milking was done only once during a lactation cycle. After the test, before being returned to their home cage, the anaesthetized lactating females were laid down in an individual cage placed on a heating plate, and looked after until complete wake up. No adverse effect of the anesthetic was noted on maternal and pup behavior: the females resumed normal maternal care (nursing, licking) and all pups were successful in sucking. The number of stimulus females used in the discrimination tests was reduced to one female for 2 litters tested. Handling and/or testing effects on newborn pups were kept minimal in designing brief tests (2 min). The number of tested pups was kept as low as possible, but sufficient to ensure statistical validity of data.

### Animals and housing conditions

Balb/c mice (*Mus musculus*, Charles River, L'Arbresle, France) were housed in standard Plexiglas cages (28×17×13 cm). Males were left with females to favor their parental contribution and ensure conception at postpartum estrous. The animals were kept under constant 12∶12 h light/dark cycle (light on at 8 a.m.) and temperature (20–22°C). Water and pellets (SAFE, Augy, France) were provided *ad libitum*. The animals were fed the same pelleted chow throughout the study, composed of 21.4% of protein, 5.1% of fat, 5.7% of ash (mineral material) and 4% fibers. Pellets were constituted from wheat, corn, wheat bran, barley, extruded soya seeds, soya meal, condensed fish soluble, yeast, calcium carbonate, dicalcium phosphate, and vitamin (A, E and D3) and oligoelement (copper sulfate pentahydrate) premixes. The breeding cages were lined with wood sawdust (SAFE, Augy, France). The number of pups by litter was culled to 6 to standardize pup competition at nursing.

### Test Animals

Neonatal mice (n = 381, from 112 litters) were tested on postnatal days 2 (P2), 6 (P6) or 15 (P15) (P2: n = 129 pups from 42 litters, P6: n = 123 from 35 litters, or P15: n = 129 from 35 litters). The day of parturition was deemed as P0. These ages were chosen because: 1) up to P17, mouse pups are exclusively suckled [58], [59], hence deriving food-related chemosensory experience from milk (no data found on the initiation of cæcotrophy in *Mus*); 2) major changes in mouse milk composition occur during the first postpartum days and between weeks 1 and 2 [18]–[22], leading to compare pup responses to milk odor during week 1 (P2 *vs.* P6 comparison) and between weeks 1 and 2 (P2–P6 *vs.* P15 comparison). Pups were tested in only one assay. To prevent litter effects, at most 5 pups of a same litter were tested in a same experiment.

### Stimuli

Fresh milk was collected from 129 unfamiliar lactating females (LF) on different days of lactation: 47, 39 and 43 on lactation days 2 (L2), 6 (L6) and 15 (L15), respectively. P2, P6, and P15 groups of pups were exposed to the L2 milk from 19, 14, and 14 LF; to the L6 milk from 13, 13, and 13 LF, and to the L15 milk from 15, 13, and 15 LF. A pup was never exposed to the milk from its own mother. The control stimulus consisted in mineral water.

Donor females were first separated from their litter for 2 h (a procedure that did not affect subsequent milk production [60]). They were anaesthetized by an intraperitoneal injection of a mixture of ketamine (Imalgène 1000, Vibrac, France; dose: 45 mg/kg in NaCl .9%) and xylazine (Rompun 2%, Bayer, Puteaux, France; dose: 5 mg/kg in NaCl .9%). The females were then injected intraperitoneally with 0,15 mL of oxytocin (Intervet, Unterschleissheim, Germany) and gently massaged on the mammary areas to stimulate milk let-down [40], [41]. Each nipple was aspired with a Pasteur pipette (2 mL). During the 15-min milking procedure, 0.5 to 1 mL of milk was collected and aliquoted into glass vessels kept on ice. The fresh milk was immediately used for the tests.

### Test devices and procedures

**Figure 3:**
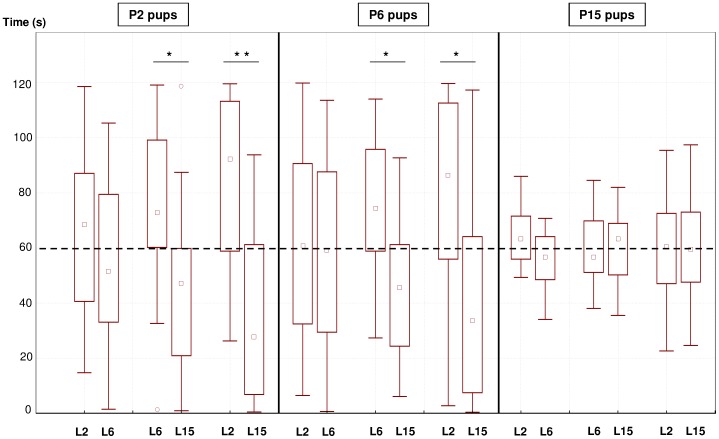
The devices used to assess differential attraction to paired odor stimuli for mouse pups aged 2, 6, and 15 days (top, middle, and bottom pictures, respectively).

Behavioral testing was run in a room adjacent to the breeding room to avoid interference with unwanted odor stimuli from conspecifics. Differential responses were quantified in a two-choice paradigm using age-adapted devices/procedures (Figure 3). Poorly mobile P2 pups were exposed to a head-orientation assay where no general exploratory movements were required, while mobile P6 and P15 pups were assayed for differential exploration in age-adapted choice arenas. For P15 pups (after eyes' opening), tests were run in red light during the dark period. For all assays, the test arena was put on a heating plate (28×20 cm; Gestigkeit, Düsseldorf, Germany) which temperature was set at 33°C. All tests were videotaped for 2 min to subsequently analyze the duration of pup orientation to, or approach of, either stimulus. These analyses were made blindly using the Observer software (Noldus, Wageningen, the Netherlands).

#### Behavioral assay for 2 day-old pups: Device

A head-orientation test was devised (adapted from [61]). Blotting paper (Tork Universal Wiper 310 Centrefeed Roll, Göteborg, Sweden) was taped on the heating plate. Two auto-adhesive labels (0.8×1.2 cm; ApliI Paper, Barcelona, Spain) were stuck bilaterally at 3 mm of the midline on the blotting paper, delimiting two equidistant areas. Twenty µL of stimulus were applied with a micropipette on each label.

#### Behavioral assay for 2 day-old pups: Procedure and dependent variable

Pups were held between the gloved thumb and index (gloves: VWR International, Leuven, Belgium) to be first approached from each olfactory sources during 6 s without contact, following a pre-test procedure used with the neonates of other species [62], [63]. Then, the test began when the body and head of the pup were aligned on the midline of the device. The pup body was held aligned during the whole test so that only cephalic motions were possible. The duration of head orientation to either stimulus was measured. An animal was considered to be oriented towards a stimulus when both of its nostrils crossed the midline of the device. Pups that did respond to this criterion were excluded from further analyses. The blotting paper and both labels were replaced after every trial.

#### Behavioral assay for 6 day-old pups: Device

It consisted in a rectangular polypropylene box (internal length×width×height: 7.1×5×7 cm) with a stainless steel mesh floor (mesh size: 3.14 mm^2^) affixed so that it was 0.7 cm above the bottom. Under the mesh, two rectangular polypropylene plates (6.1×2.1 cm, 0.2 cm thickness) were inserted in two equal compartments (separated by a polypropylene barrier to avoid the mixing of odors). Forty µL of milk were spiked in standardized fashion on each plate placed to 2 mm under the mesh.

#### Behavioral assay for 6 day-old pups: Procedure and dependent variable

Before testing, the pups were introduced for 6 s into each part of the arena separated by a polypropylene barrier. The order of this stimulus pre-presentation was at first random, and then counterbalanced at each other test. For the test itself, the pup was placed on the midline of the arena, and left free to move. The duration of exploratory roaming in the two halves of the arena was recorded. A pup was considered to be in one half of the arena when its entire muzzle crossed the midline. Pups that performed less than 3 midline crossings or that urinated were excluded from further analyses. All elements of the device were thoroughly washed with 95° ethyl alcohol and distilled water, and then dried.

#### Behavioral assay for 15 day-old pups: Device

The arena consisted in a rectangular polycarbonate box (16×11×13.5 cm) with a stainless steel wire-mesh (mesh size: 3.14 mm^2^) placed 0.7 cm above the bottom. Under the mesh, two equal compartments were delimited by a polypropylene barrier. Rectangular polypropylene plates (7.8×11 cm, 0.2 cm thickness) carrying the stimuli were inserted in each compartment. Sixty µL of milk were spiked in a standardized pattern on each plate and placed at a distance of 2 mm under the mesh.

#### Behavioral assay for 15 day-old pups: Procedure and dependent variable

The procedure described for 6 day-old pups was repeated here.

### Experimental groups

Eighteen experimental groups were formed. First, we assessed whether the odor of L2, L6 and L15 milk could be differentiated from water by P2, P6 and P15 pups. Three series of tests were run at each age opposing a milk sample and the control stimulus [L2 milk vs. water (P2 pups: n tested/n analyzed = 26/20, P6: n = 28/20, and P15: n = 20/20; from 9, 5, and 6 litters, respectively); L6 milk vs. water (P2: n = 22/20, P6: n = 22/18, and P15: n = 22/22; from 5, 5, and 7 litters, respectively), and L15 milk vs. water (P2: n = 33/25, P6: n = 24/18, and P15: n = 24/24; from 7, 6 and 6 litters, respectively)].

Second, we examined whether different milk would convey more or less olfactory potency for P2, P6 and P15 pups according to the period of milk collection during lactation. Three series of tests were performed at each age contrasting the odors of two milk samples [L2 vs. L6 milk (P2 pups: n tested/n analyzed = 31/22, P6: n = 22/21, and P15: n = 20/20; from 6, 5, and 5 litters, respectively); 5) L6 vs. L15 milk (P2: n = 27/21, P6: n = 25/20, and P15: n = 20/20; from 7, 5, and 5 litters), and 6) L2 vs. L15 milk (P2: n = 30/21, P6: n = 34/26, and P15: n = 23/23; from 8, 9, and 6 litters, respectively)].

### Statistical Analyses

The Shapiro-Wilks test indicating non-normal distribution of data in most groups, non-parametric statistics were used (Statistica 8, Statsoft, Paris, France). Thus, Wilcoxon tests were applied to compare the total time pups spent oriented to either side of the choice devices. Kruskal-Wallis tests yielded no significant litter effect (0.90<H<10.88, in all cases, p>0.05) and no significant milk donor effect in any group (0.03<H<9.70, in all cases, p>0.05).
